# Pad in forensic psychiatry

**DOI:** 10.1192/j.eurpsy.2021.115

**Published:** 2021-08-13

**Authors:** I. Franke

**Affiliations:** Forensic Psychiatry And Psychotherapy, Ulm University, Guenzburg, Germany

**Keywords:** PAD, prison psychiatry, ethics, decision-making

## Abstract

**Introduction:**

A recent court decision in Germany defined assisted suicide as a basic human right. Consequently, the discussion regarding PAD needs to be extended to people who are in forensic/secure psychiatric hospitals or prisons, sometimes without any prospects of release. Several studies have shown that long-term hospitalization and detention are associated with feelings of hopelessness, depression and suicidal ideations. Moreover, the resources for adequate therapy are often rare. This results in complex moral challenges for mental health care.

**Objectives:**

To review current practices in countries that allow PAD and to discuss ethical conflicts.

**Methods:**

Literature review; international comparison of current regulations.

**Results:**

A majority of the literature on PAD in detention refers to prisoners with terminal medical conditions. Single case reports of PAD-requests of mentally disordered offenders aroused great public interest. The resulting ethical conflicts are similar to those issues regarding PAD and mental disorder in general. However, in secure treatment settings and detention additional aspects such as adverse living conditions and inadequate access to mental health care need to be taken into account.

**Conclusions:**

If unbearable pain is not a precondition for assisted suicide, then mentally disordered and healthy offenders have a right to request PAD, provided they have medical decision-making capacity. Considering the common insufficient mental health care for people in detention, policy and law makers need to ensure that access to PAD will not replace therapy. Professionals involved in PAD evaluations need support by specific guidelines.

**Disclosure:**

No significant relationships.

